# Trends of adverse events and mortality after DMARDs in patients with rheumatoid arthritis: Interrupted time‐series analysis

**DOI:** 10.1002/iid3.630

**Published:** 2022-06-20

**Authors:** Yao‐Fan Fang, Jia‐Rou Liu, Shu‐Hao Chang, Chang‐Fu Kuo, Lai‐Chu See

**Affiliations:** ^1^ Division of Rheumatology, Allergy and Immunology, Department of Internal Medicine Chang Gung Memorial Hospital at Linkou Taoyuan City Taiwan; ^2^ Department of Public Health, College of Medicine Chang Gung University Taoyuan City Taiwan; ^3^ Biostatistics Core Laboratory, Molecular Medicine Research Centre Chang Gung University Taoyuan City Taiwan

**Keywords:** diseases, rheumatoid arthritis

## Abstract

**Objectives:**

Patients with rheumatoid arthritis (RA) experience adverse events because of the characteristics of the disease and the side effects of medications. We investigated the trends of adverse events and mortality associated with disease‐modifying antirheumatic drugs (DMARDs).

**Methods:**

We used the Taiwan National Health Insurance Database to enroll patients with incident RA between 2000 and 2017. The 1‐year incident rate of gastrointestinal (GI) bleeding and 3‐year incident rates of other adverse events and mortality for each calendar‐quarter cohort were computed and adjusted using propensity score‐based stabilized weights for fair comparisons. Levels and trends of the conventional DMARD era (2000–2002, Phase 1) were compared with those of the TNFi era (2003–2012, Phase 2) and OMA era (2013–2017, Phase 3) by using interrupted time series (ITS) analysis.

**Results:**

All patients with RA were prescribed cDMARDs in Phase 1 (2000–2002), and 1%–3% were prescribed either TNFi in phase 2 (2003–2012) or OMAs in phase 3 (2013–2017). The cancer incidence rate was 1.90%, and its mortality rate was 4.19%. After the introduction of TNFi from 2003 to 2012, the main outcomes, except TKA, exhibited a steady or mild decrease in trends. ITS analysis revealed that the slope mildly increased in 2003–2012 compared with that in 2000–2003 by 0.13% for total knee replacement (*p* = .0322). In 2012–2017 (the OMA era), the events became steady.

**Conclusion:**

In patients with RA, the introduction of DMARDs was associated with stable adverse events and mortality rates. Moreover, the introduced new treatment for RA exhibited a good safety profile.

## Graphical abstract

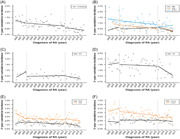

## 1 INTRODUCTION

Rheumatoid arthritis (RA), an autoimmune and systemic disease, requires continual medication to control the disease activity and prevent progressive disability. However, the long‐term use of mediation and the character of the disease place patients with RA at increased risks of gastrointestinal (GI) bleeding, major adverse cardiac events (MACEs), overall venous thromboembolism (OVT), tuberculosis (TB), cancer, joint replacement, and mortality.^1^, ^2^, ^3^, ^4^, ^5^, ^6^ Conventional disease‐modifying antirheumatic drugs (cDMARDs), such as hydroxychloroquine, methotrexate, leflunomide, sulfasalazine, cyclosporine, azathioprine, and D‐penicillamine, were the mainstay treatment before biologic disease‐modifying antirheumatic drugs (bDMARDs). Several clinical trials and real‐world evidence have demonstrated that newly introduced bDMARDs are associated with increased risks of adverse events.^7^, ^8^, ^9^, ^10^, ^11^, ^12^ If RA treatment with cDMARDs and bDMARDs exhibited stable or decreased risks of GI bleeding, MACE, OVT, TB, cancer, joint replacement, and mortality, the safety and effectiveness of these drugs could be balanced in our daily practice. Nevertheless, the EULAR guideline suggests early and intensive intervention with DMARDs for patients with RA to prevent progressive joint damage.^13^ The safety profiles of DMARDs in patients with RA in clinical trials and observational studies are controversial. In particular, the effects of these medications on MACE, OVT, and cancer have been seldom studied in randomized controlled trials (RCTs) or observational studies. Furthermore, patients in RCTs have been typically carefully selected, and the results of observational studies may not be generalizable to all patients with RA because bDMARDs are affordable only to those with a high income and/or health insurance.

In Taiwan, the National Health Insurance (NHI) program was established in 1995 and entails compulsory enrollment by the population of Taiwan. The coverage rate is therefore exceptionally high—99.5%. Furthermore, patients with RA are eligible for catastrophic illness certificates, which entails waiver a of outpatient and inpatient copayments. The Taiwan NHI program reimburses various bDMARDs, including tumor necrosis factor inhibitor (TNFi), anti‐CD20 (rituximab), T‐cell costimulatory inhibitor (abatacept), anti‐IL‐6 (tocilizumab), and Janus kinase inhibitors (JAKis). The first antitumor necrosis factor, etanercept, was initially reimbursable on March 1, 2003. T‐cell costimulatory inhibitor and anti‐IL‐6 reimbursement were introduced on May 1, 2012, and JAKi (tofacitinib) was initially reimbursable on December 1, 2014. Hence, the Taiwan NHI database is an excellent data source to examine DMARD use and its safety profile.

In this study, we performed an interrupted time‐series (ITS) analysis to investigate the use of RA medication and its safety profile in patients with RA from 2000 to 2017 using the NHI database. The three phases were defined: phase 1 from 2000–2002 (cDMARD era), phase 2 from 2003–2012 (TNFi era), and phase 3 from 2013–2017 (OMA era). The outcome statistics were the 1‐year incidence rate of GI bleeding; 3‐year incidence rates of acute myocardial infarction (AMI), congestive heart failure (CHF), ischemic stroke/transient ischemic attack (IS/TIA), overall thromboembolism (OVT; comprising pulmonary thromboembolism and deep vein thrombosis), tuberculosis (TB), total hip replacement (THR), total knee replacement (TKR), cancer, and all‐cause mortality. Reasons for using the ITS analysis were as follows: (1) use of observational data in an aggregate format and presentation as graphical results, facilitating examination of the time trend (including level change and slope change) of practice (patient volume or medication prescription) and outcome statistics before and after the introduction of a new medication (namely “interruption”) and (2) minimized selection bias because every individual is considered in the calculation of outcome statistics.^14^

## 2 PATIENTS AND METHODS

### 2.1 Data sources

Our primary data source was the Taiwan NHI database and Taiwan Death Registry (TDR).^15^ The NHI database contains detailed information on patient registration and original claims data, including demographics (such as year of birth, place of residence, and insurance details) and outpatient or inpatient visit (dates of inpatient and outpatient visits, medical diagnostic codes and prescriptions details, medical expenditures, examination code, operation or procedure code, and discharge status). The TDR contains the dates of death and underlying causes of death of the deceased residents of Taiwan. The completeness of the TDR and the accuracy of the cause‐of‐death coding in Taiwan are well recognized.^16^ A unique personal identifier assigned to each Taiwan resident allows for linkage between the NHI database and TDR. The unique personal identifiers are encrypted before data are released to researchers to ensure confidentiality. The NHI database and TDR are available at the Health Welfare Data Science Center or its subcenters for further privacy protection. Only summarized results can be taken from the center, not individual data. The diagnostic coding system in the NHI database follows the *International Classification of Diseases, Nineth [Tenth] Revision, Clinical Modification* (*ICD‐9‐CM* from 2000 to 2015 and *ICD‐10‐CM* from 2016 to present). The NHI database's validity, representativeness, and clinical consistency have been described elsewhere.^17^

The Institutional Review Board of Chang Gung Medical Foundation (201901028B1) exempted the need for informed consent from participants in this study because the original identification numbers of each patient in the NHI database and TDR were encrypted.

### 2.2 Study design and study population

This was a nationwide ITS analysis. We identified patients with newly diagnosed RA (*ICD‐9‐CM*: 714.0 and *ICD‐10‐CM*: M05‐06) from 2000 to 2017. Only patients with RA possessing a catastrophic illness certificate were enrolled to reduce RA misclassification. In Taiwan, the issuance of this certificate requires physicians to apply along with all evidence supporting the disease diagnosis. The certificate is issued after a formal review by expert panels commissioned by the NHI Administration. A review panel used both the 1987 and 2010 classification criteria for RA to evaluate the validity of RA. The index date was defined as the date of newly diagnosed RA. Patients who were <20 or >80 years old at the index date were excluded. Patients with missing sex data (*n* = 408), incorrect data (date of death before index date, *n* = 1), or no DMARD treatment after RA diagnosis (*n* = 3083) were excluded.

Next, we formed 72 cohorts based on the calendar quarter of the index date. Hence, Phases 1 (2000–2002), 2 (2003–2012), and 3 (2013–2017) had 12, 40, and 20 cohorts, respectively. Finally, we compared the 1‐year incidence rates of GI bleeding and 3‐year incidence rates of MACE, OVT, TB, cancer, joint replacement, and mortality among three phases to investigate whether the adverse events were associated with the use of cDMARDs, TNFi and OMA in Taiwan.

### 2.3 Intervention

Patients with RA who used bDMARDs were defined as biologic‐näive patients. The cDMARD era was from 2000 to 2002 (Phase 1). Etanercept was first available for RA treatment in Taiwan in 2003, and other TNFis were introduced successively until 2012. Therefore, we called the years from 2003 to 2012 the TNFi era (Phase 2). Other‐mechanism agents (OMAs), such as abatacept, tocilizumab, and tofacitinib, were introduced after 2012. Hence, the period from 2013 onward is the OMA era (Phase 3). Of note, TNFi use drastically increased in 2003 in Taiwan.^18^ Moreover, in 2012, two other‐mechanism agents were available for RA treatment in Taiwan.^19^ We calculated the 1‐year (or 3‐year) distribution of different RA medications for each quarter‐cohort as the sum of prescription days for one of the RA medications divided by the sum of prescription days for all RA medications in 1 year (or 3 years).

### 2.4 Outcomes

The study outcomes were GI bleeding, AMI, CHF, IS/TIA, OVT, TB, THR, TKR, cancer, and all‐cause mortality. Only a primary discharge diagnosis of GI bleeding, AMI, CHF, IS/TIA, OVT, THR, and TKR was considered to prevent misclassification of study outcomes. In addition to *ICD‐9‐CM* or *ICD‐10‐CM* codes, the catastrophic illness certificate for cancer was used to identify patients with cancer (*ICD‐9‐CM* codes 140–208 and *ICD‐10_CM* codes C; Table S1). Mortality was based on the TDR. We calculated the 1‐year GI bleeding rate for each quarter cohort as the number of GI bleeding incidences within 1 year after RA diagnosis divided by the total number of patients with newly diagnosed GI in each quarter cohort. For other outcomes, we computed 3‐year rates.

### 2.5 Covariates

Other than age and sex, the comorbidity of interest was cancer history, DM, hypertension, clinical kidney disease (CKD). The above comorbidities had to appear at least twice in outpatient visit or at least once in hospitalization (Table S1).

### 2.6 Statistical analysis

We plotted the percentages (%) of cDMARDs, TNFi, and OMAs used, the proportion of baseline characteristics (such as age, sex, comorbidities, and medication use) outcome statistics versus calendar time.

All outcome statistics across each cohort were adjusted using propensity score‐based stabilized weights (PSSWs) to ensure age, sex, and comorbidities were well‐balanced. The advantages of PSSWs are preserving the sample size of each cohort and provision of a pseudo‐population.^20^ We used a generalized boosted model (GBM) to estimate PSSWs to achieve optimal balance among each cohort. A GBM is optimal for selecting the best mathematical form (including interactions) of covariates and is not sensitive to outlying weights.^21^ The covariates in Section 2.5 were included in the GBM. We used the absolute standardized mean difference (ASMD) to assess the comparability of covariates between cohorts, and the threshold of 0.1 for ASMD implies that the difference between covariates was at most 0.05% among the various cohorts.^22^

We used ITS to estimate the study outcomes' level change and slope change between Phases 2 and 3. In ITS, we fitted the segmented least‐squares regression lines of the outcome variable (*Y*), time (*T*), phase 2 (*X*_2_), and Phase 3 (*X*_3_); K1 and K2 are the two inflection points for the regression as follows (Supplemental Figure S1):

$$Y_{T}=\beta_{0}+\beta_{1}T+\beta_{2}X_{2}+\beta_{3}(T–K_{1})+\beta_{4}X_{3}+\beta_{5}\left(T\;–\;{\text{K}}_{\text{2}}\right)+\text{error}.$$

*Y*_*T*_ represents the statistics of a study outcome and was adjusted after using the PSSW, with each equally spaced time point *T* (outcome section). *T* represents the time elapsed since the start of the study (from 0, 1, 2, …, to the end of the last time unit). For example, (*T*–*K*_1_) was the time elapsed since Phase 2 (from 0, 1, 2, …, to the end of the last time unit). Similarly, (*T*−*K*_2_) was the time elapsed since Phase 3 (from 0, 1, 2, …, to the end of the last time unit). *X*_2_ was given a value of 1 for Phase 2 and 0 otherwise. *X*_3_ was given a value of 1 for Phase 3 and 0 otherwise.

The coefficient of *β*_0_ represented the level of the outcome rate at *T* = 0, and *β*_1_ indicated the slope trend in Phase 1. The coefficients for *β*_2_ and *β*_3_ represented the level and slope changes from Phase 1 to Phase 2. The coefficients for *β*_4_ and *β*_5_ represented the level change and slope change from Phase 2 to Phase 3, respectively. Statistical tests of *β_i_* (estimate of *β_i_* divided by its standard error, where *i* = 1 to 5) were used to examine their effects.

## 3 RESULTS

### 3.1 Baseline characteristics

**Figure 1:**
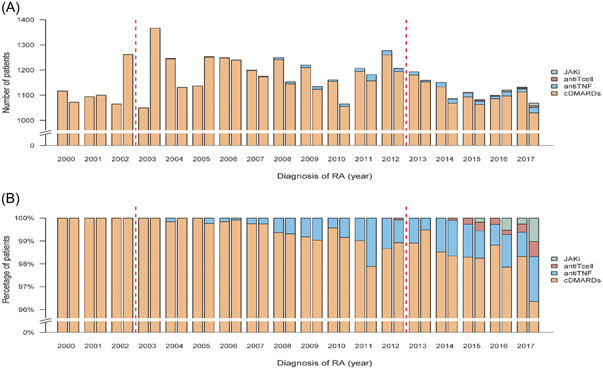
Patients number, distribution of RA medication by half‐year period from 2000 till 2017, Taiwan. (A) The number of new RA patients treated with RA medication, (B) The distribution of RA medication use based on 1‐year cumulative daily prescription. cDMARD, conventional disease‐modifying antirheumatic drug; JAKi, Janus kinase inhibitor; RA, rheumatoid arthritis; TNF, tumor necrosis factor.

We identified 47,487 patients with incident RA diagnosed between January 1, 2000, and December 31, 2017. After excluding patients with missing data, patients who died before the index date, and patients with prescriptions for RA medications before the index date, 41,821 patients with RA were eligible for analysis. In addition, we identified patients with RA who used cDMARDs and bDMARDs for a half‐year period. There were about 1000 to 1300 patients every half year during 2000 to 2017. Regarding the RA medication, cDMARDs were predominately prescribed for RA patients during the entire study duration, bDMARDs slowly increased from less than 1% in 2003 to 4% in 2017 (Figure 1).

**Figure 2:**
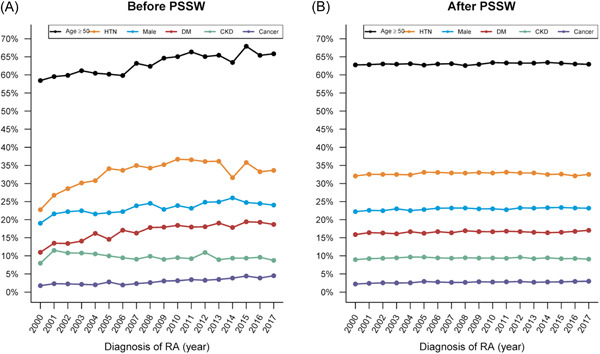
Sex, age, and comorbidities among patients with RA by half‐year period from 2000 till 2017, Taiwan. (A) before and (B) after PSSW. CKD, chronic kidney disease; DM, diabetes mellitus; HTN, hypertension; PSSW, propensity score‐based stabilized weight; RA, rheumatoid arthritis.

Figure 2 describes the percentage of aged ≥50 years, men, and different comorbidities between 2000 and 2017. The proportion of men (approximately 20%) was stable from 2000 to 2017, but the proportion of patients aged ≥50 years increased from 59% in 2000 to 66% in 2017. The prevalence rates of cancer (2% → 5%), diabetes mellitus (10% → 16%), and hypertension (22% → 31%) increased over time, but that of chronic kidney disease (roughly 10%) remained stable. After PSSWs, the proportions of men, patients aged >50, and the prevalence rate of comorbidities were similar over time (Figure 2).

### 3.2 GI bleeding

**Figure 3:**
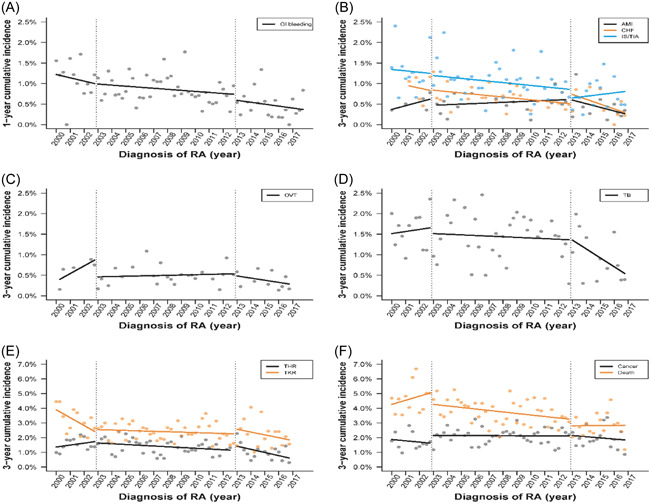
(A–F) Trends of adverse events and mortality after biological disease‐modifying antirheumatic drugs in patients with rheumatoid arthritis: interrupted time‐series analysis. AMI, acute myocardial infarction; CHF, congestive heart failure; GI, gastrointestinal; IS, ischemic stroke; OVT, overall venous thromboembolism; RA, rheumatoid arthritis; TB, tuberculosis; THR, total hip replacement; TIA, transient ischemic attack; TKR, total knee replacement.

Figure 3A presents the 1‐year incidence rate of GI bleeding from 2000 to 2017. In Phase 1, the 1‐year incidence rate of GI bleeding in 2000 was 1.22% and decreased by 0.02% per quarter (*p* = .4940). In Phase 2, the level or slope of the incidence rate was similar to that of Phase 1 (+0.01%, *p* = .9771; +0.01% per quarter, *p* = .6452, respectively). Similarly, the level or slope of incidence rate was similar between Phases 2 and 3 (−0.14%, *p* = .4511; −0.01% per quarter, *p* = .7087; Table 1).

**Table 1:** Changes in the cumulative incidence rate of adverse events after PSSW based on interrupted time series analysis.

		Level of Phase 1 (***β***_0_)	Slope of Phase 1 (***β***_1_)	Level diff of Phases 1 and 2 (***β*_2_**)	Slope diff of Phases 1 and 2 (***β*_3_**)	Level diff of Phases 2 and 3 (***β*_4_**)	Slope diff of Phases 2 and 3 (***β*_5_**)
GI bleeding	Mean ± SE	1.24 ± 0.21%	−0.02 ± 0.03%	−0.01 ± 0.22%	0.01 ± 0.03%	−0.14% ± 0.19%	−0.01% ± 0.01%
	*p* value	<.0001***	.4940	.9757	.6452	.4848	.7087
AMI	Mean ± SE	0.35 ± 0.25%	0.02 ± 0.03%	−0.15 ± 0.21%	−0.02 ± 0.03%	0.01 ± 0.21%	−0.03% ± 0.02%
	*p* value	.1796	.4373	.4671	.5195	.9702	.1387
CHF	Mean ± SE	1.05 ± 0.57%	−0.02 ± 0.06%	0.01 ± 0.25%	0.01 ± 0.06%	0.25 ± 0.22%	−0.02 ± 0.02%
	*p* value	.0725	.7703	.9618	.8751	.2616	.2562
IS/TIA	Mean ± SE	1.35 ± 0.27%	−0.01 ± 0.04%	−0.05 ± 0.29%	0.00 ± 0.04%	−0.22 ± 0.26%	0.02 ± 0.02%
	*p* value	<.0001***	.8217	.8786	.9983	.3980	.4336
OVT	Mean ± SE	0.31 ± 0.19%	0.05 ± 0.02%	−0.41 ± 0.19%	−0.05 ± 0.02%	−0.04 ± 0.17%	−0.02 ± 0.01%
	*p* value	.1178	.0612	.0349*	.0753	.8243	.3037
TB	Mean ± SE	1.51 ± 0.33%	0.01 ± 0.05%	−0.14 ± 0.35%	−0.02 ± 0.05%	0.04 ± 0.35%	−0.05 ± 0.03%
	*p* value	<.0001***	.7767	.7009	.7166	.9016	.1136
THR	Mean ± SE	1.33 ± 0.28%	0.03 ± 0.04%	−0.08 ± 0.29%	−0.05 ± 0.04%	0.33 ± 0.29%	−0.04 ± 0.03%
	*p* value	<.0001**	.3983	.7953	.2490	.2575	.1221
TKR	Mean ± SE	4.03 ± 0.42%	−0.13 ± 0.06%	0.13 ± 0.43%	0.13 ± 0.06%	0.35 ± 0.42%	−0.04 ± 0.04%
	*p* value	<.0001***	.0221*	.7660	.0322*	.4018	.2943
Cancer	Mean ± SE	1.90 ± 0.37%	−0.02 ± 0.05%	0.51 ± 0.37%	0.02 ± 0.05%	0.04 ± 0.36%	−0.02 ± 0.03%
	*p* value	<.0001***	.6579	.1736	.6732	.9083	.5757
Death	Mean ± SE	4.19 ± 0.51%	0.07 ± 0.07%	−0.75 ± 0.52%	−0.10 ± 0.07%	−0.46 ± 0.50%	0.03 ± 0.05%
	*p* value	<.0001***	.3122	.1560	.1741	.3666	.5441

Abbreviations: AMI, acute myocardial infarction; CHF, congestive heart failure; GI, gastrointestinal; IS, ischemic stroke; OVT, overall venous thromboembolism; PSSW, propensity score‐based stabilized weight; diff, difference; SE, standard error; TB, tuberculosis; TIA, transient ischemic attack; THR, total hip replacement; TKR, total knee replacement.

* *p* < .05

** *p* < .01

*** *p* < .001.

### 3.3 Major cardiac events

Figure 3B shows the 3‐year incidence rates of AMI, CHF, and IS/TIA. In Phase 1, the 3‐year rates of AMI, CHF, and IS/TIA in were 0.37%, 1.03%, and 1.34%, respectively. The ITS analysis revealed that neither the level nor slope change in these major cardiac events between Phases 1 and 2, and Phases 2 and 3 reached statistical significance (Table 1).

### 3.4 Overall thromboembolism

Figure 3C shows the 3‐year incidence rate of OVT from 2000 to 2017. The 3‐year rate of OVT was 0.35% in 2000. The ITS analysis demonstrated a significant drop in the OVT rate from Phase 1 to Phase 2 (−0.45%, *p* = .0310), and a marginal difference was observed in the slope difference between Phases 2 and 1 (−0.04%, *p* = .0753). However, no significant difference in level and slope between Phases 2 and 3 was observed (Table 1).

### 3.5 Tuberculosis

Figure 3D shows the 3‐year incidence rate of TB from 2000 to 2017. In 2000, the 3‐year rate of TB was 1.51%. The trend of TB incidence rate started to decrease in Phase 2, and continued to decrease in Phase 3. The ITS analysis revealed that neither the level difference nor the slope difference statistical significance between Phases 1 and 2, and between Phases 2 and 3 (Table 1).

### 3.6 Joint replacement rate

Figure 3E shows the 3‐year incidence rate of THR and TKR from 2000 to 2017. For THR, the 3‐year incidence rate was 1.37% in 2000. No significant difference was observed in the level change and slope change of the 3‐year incidence rate between Phases 2 and 1 and between Phases 3 and 2. For TKR, the 3‐year incidence rate was 3.89%. The slope of TKR exhibited a significant decrease in Phase 1 (−0.13% per quarter, *p* = .0221). The slope in Phase 2 significantly increased compared with that in Phase 1 (0.13% per quarter, *p* = .0323). Furthermore, no difference was observed in the level and slope between Phases 2 and 3 (0.31%, *p* = .4323; −0.04% per quarter, *p* = .2943; Table 1).

### 3.7 Cancer and all‐cause mortality

Figure 3F shows the 3‐year incidence rate of cancer and all‐cause mortality from 2000 to 2017. The 3‐year cancer rate and 3‐year mortality rate in 2000 was 1.87% and 4.26%, respectively. The trend of both the cancer rate and mortality mildly decreased and remained stable in Phases 1–3 (Table 1).

## 4 DISCUSSION

In this nationwide ITS study, we identified the time trend of GI bleeding, MACE, OVT, TB, joint replacement rates, cancer, and mortality after the introduction of cDMARDs (Phase 1), TNFi (Phase 2), and OMAs (Phase 3). We observed that the 1‐year GI bleeding rate started to decrease after cDMARD treatment was introduced and remained stable after bDMARDs. The 3‐year incidence of MACE, OVT, TB, cancer, and mortality remained steady and mildly decreased after introducing cDMARDs and bDMARDs. However, TKR started to decrease among patients with RA after cDMARD treatment became available and the slope significantly increased after TNFi introduction, whereas the incidence rate of THR exhibited a steady trend after both cDMARDs and bDMARDs treatment.

One key strength of our analysis is to represent the nationwide practice of prescribing cDMARDs, TNFi, and OMAs. In Taiwan, all patients with RA were prescribed cDMARDs in Phase 1 (2000–2002), and 1%–3% were prescribed either bDMARDs or OMAs in Phase 2 (2003–2012) and Phase 3 (2013–2017). The advantages and disadvantages of cDMARDs and bDMARDs reported previously under clinical trials or meta‐analyses were limited by population or well‐organized planning, respectively.

Our data revealed the safety trends in the nationwide use of cDMARDs and bDMARDs. No major changes were observed after introducing the various RA treatments, except in TKR after cDMARD treatment was made available, but the slope remained stable after bDMARD treatment. Studies have reported high risks of GI bleeding, MACE, OVT, TB, joint replacement, cancer, and mortality in patients with RA.^5^, ^23^, ^24^, ^25^, ^26^, ^27^, ^28^, ^29^ Our study determined that incidence rates of GI bleeding, MACE, cancer, and mortality among patients with RA were similar to those in clinical trial studies and meta‐analyses.^11^, ^30^ We consider GI bleeding to decrease after cDMARDs and bDMARDs treatment. We could taper NSAIDs medication use after disease activity is stable. However, the risk of GI bleeding was started to decrease but not obvious. One possible explanation is that selective NSAIDs, such as Celebrex, were used in 2006 in Taiwan. The risk of hospitalization GI bleeding was decreased after selective NSAIDs.^31^

CHF could be a special adverse event for introducing TNFi. An early case report demonstrated new‐onset heart failure.^32^ Another study reported no difference in new‐onset heart failure in RA patients with TNFi.^33^ Our study has the consistent result of no increase in heart failure.

Regarding cancer incidence, our previous study revealed no overall increased risks of all types of cancer in RA patients.^34^ This study further proved the stable cancer risk after different mechanism of RA treatment. However, long‐term follow‐up duration may be needed.

One study suggested an increased risk of OVT in patients with RA in the literature.^25^ Moreover, patients with RA with JAKi exhibited an increased risk of OVT in clinical trials.^31^, ^32^, ^33^ Our study identified a low incidence of OVT, and the rate did not increase after OMA introduction. This may be explained by a low OVT risk in Asian patients.

The literature has also revealed an increased risk of TB incidence after the introduction of TNFi.^34^ However, our study indicated a continued decrease in the incidence rate of TB. The reason may be that our local guideline suggests using a risk management plan before TNFi treatment.

The incidence rate of TKR decreased after cDMARD use and mildly increased after TNFi use. Some studies also concerned the severe infection in RA patients with TNFi.^38^ Another study reported the increased post‐operation infection risk in RA patients with TNFi.^39^ Increased risk of TKR after TNFi treatment should be monitored, but further research may be needed to prove the concept. The rate of THR remained stable with the use of cDMARDs and bDMARDs in our study. Cordtz et al. suggested that the introduction of bDMARDs was associated with a decreased incidence rate of TKR, whereas the incidence of THR started to decrease before bDMARD introduction.^35^

Our study has some limitations. First, misclassification of patients with RA is possible when performed based on *ICD* codes. However, this risk was minimal because of the strict review of RA diagnoses before issuing a catastrophic illness certificate. The duration and dose of bDMARDs or cDMARDs is another concern. To our clinical knowledge, most RA patients in Taiwan tak standard dose cDMARDs and bDMARDs. Second, few patients used TNFi and OMAs in Phases 2 and 3. Third, other than the RA drugs, the patient's habits (such as cigarette smoking, alcohol consumption, and exercise) are associated with the study outcomes. Unfortunately, patient habits are not available in the NHIRD. Fourth, other drugs might be associated with the study outcomes as well, including oral corticosteroids, non steroidal anti‐inflammatory (NSAID), antihypertensive, hypoglycemic, and anti‐coagulant/anti‐platelet. As the prescription rate of the above medications were highly correlated with the comorbidities, we did not put it as covariates. Fifth, the NHIRD does not contain patients' detailed laboratory results, such as erythrocyte sedimentation rate, C‐reactive protein, disease activity scores, and disease stage.

In conclusion, we determined that the evolutions of GI bleeding, MACE, OVT, TB, cancer, joint replacement rates, and mortality were generally steady and mildly decreased after the introduction of cDMARDs and bDMARDs. In addition, the risks of adverse events and mortality did not increase after RA treatment.

## AUTHOR CONTRIBUTIONS

*Design*: Yao‐Fan Fang, Chang‐Fu Kuo and Lai‐Chu See. *Data collection and analysis*: Jia‐Rou Liu and Shu‐Hao Chang. *Drafting of manuscript*: Yao‐Fan Fang and Lai‐Chu See. *Manuscript revision and approval*: all authors.

## CONFLICT OF INTEREST

The authors declare no conflict of interest.

## ETHICS STATEMENT

The Institutional Review Board of Chang Gung Medical Foundation (201901028B1) exempted the need for informed consent from participants in this study because it had the lowest risk of personal information leakage and the original identification numbers of each patient in the NHI database and TDR were encrypted.

## Supporting information

Supporting information.

Supplementary Information
