# Supplementary material for: Trends of adverse events and mortality after DMARDs in patients with rheumatoid arthritis: Interrupted time‐series analysis
Source: Immun Inflamm Dis. 2022 Jun 20;10(7):e630. doi: 10.1002/iid3.630 (PMC9208285; doi:10.1002/iid3.630)
Supplement: Supplementary file 1 — Supporting information. [file IID3-10-e630-s002.docx]

**Table S1.** Codes for comorbidities and outcomes

|  | **International Classification of Disease 9th revision (ICD-9) code** | **International Classification of Disease 10th revision (ICD-10) code** |
| --- | --- | --- |
| **Comorbidity** |  |  |
| Hypertension | 401, 402 | I10-I16 |
| Diabetes | 250, 249 | E08, E09, E11, E13 |
| Chronic kidney disease | 580-589 | I12, I13, N00-N05, N07, N11, N14, N17-N19, Q61 |
| Cancer | 140-208 | C |
| **Outcomes** |  |  |
| GI bleeding | 455.2, 455.5, 455.8, 456.0, 456.2, 530.7, 530.82, 531.0-531.6, 532.0-532.6, 533.0-533.6, 534.0-534.6, 535.0-535.6, 537.83, 562.02, 562.03, 562.12, 562.13, 568.81, 569.3, 569.85, 578.0, 578.1, 578.9 | K22.6, K25.0, K25.2, K25.4, K25.6, K26.0, K26.2, K26.4, K26.6, K27.0, K27.2, K27.4, K27.6, K28.0, K28.2, K28.4, K28.6, K29.0, K62.5, K92.0, K92.1, K92.2 |
| Ischemic stroke | 433, 434, 436 | I63, I64 |
| Transient ischemic attack | 435 | G45 |
| Overall venous thromboembolism | 415.1, 416.2, 453 | I26, I27.82, I80, I82 |
| Tuberculosis* | 010-018 | A15-A19 |

* The definition of tuberculosis (TB): First, the ICD-9 codes were 010-018 and the ICD-10 codes were A15-19. Second, >2 mycobacteria laboratory examination codes were used for TB identification, including those identified by acid-fast smear, mycobacterial culture, acid-fast bacillus differentiation, tuberculin test, tuberculosis test, and bronchoscopy results. Third, we identified patients who had taken at least 1 prescription consisting of >3 anti-TB drugs used simultaneously for at least 120 days during a 180-day period; these drugs were isoniazid, ethambutol, rifampin, pyrazinamide, amikacin, kanamycin, streptomycin, ciprofloxacin, ofloxacin, moxifloxacin, levofloxacin, prothionamide, clarithromycin, and thioridazine.

**Table S2.** Codes for medications

|  | **Anatomical Therapeutic Chemical (ATC) codes** |
| --- | --- |
| **cDMARDs** |  |
| Hydroxychloroquine | P01BA02 |
| Sulfasalazine | A07EC01 |
| Methotrexate | L01BA01 |
| Leflunomide | L04AA13 |
| Cyclosporine A | L04AD01, S01XA18 |
| Azathioprine | L04AX01 |
| D-penicillamine | M01CC01 |
| **TNFi** |  |
| Etanercept | L04AB01 |
| Adalimumab | L04AB04 |
| Golimumab | L04AB06 |
| **OMAs** |  |
| Abatacept | L04AA24 |
| Tofacitinib | L04AA29 |
| **Steroid** | H02AB04, H02AB06 |
| **Anti-hypertensive medications** |  |
| ACEi or ARB | C09AA01-C09AA09, C09AA16, C09BA01, C09BA02, C09BA04, C09BB, C09BB04, C09BB05, C09CA01-C09CA04, C09CA06-C09CA09, C09DA01, C09DA03, C09DA04, C09DA06-C09DA09, C09DB01, C09DB02, C09DB04, C09DB07, C09DX01 |
| Beta-blockers | A09AA02, A14AA02, C07AA01, C07AA02, C07AA03, C07AA05, C07AA06, C07AA07, C07AA12, C07AA15, C07AA19, C07AB02-C07AB05, C07AB07, C07AB09, C07AB12, C07AG01, C07BA68, C07BB02, C07BB03, C07CA03, C07DA06, R03CA91, S01ED01-S01ED05, S01ED51, S01ED54 |
| **Hypoglycemic medications** |  |
| Metformin | A10BA02 |
| Sulfonylurea | A10BB01, A10BB04, A10BB07-A10BB09, A10BB12, A10BD, A10BD02 |
| Insulin | A10AB01, A10AB03-A10AB06, A10AB30, A10AC01, A10AC03, A10AC30, A10AD01, A10AD03, A10AD05, A10AE01, A10AE04, A10AE05 |
| Thiazolidinedione | A10BG02, A10BG03 |
| DDP4-inhibitor | A10BD07, A10BD08, A10BD13, A10BH01-A10BH05 |
| SGLT2 inhibitor | A10BD15, A10BD20, A10BK01, A10BK02, A10BK03 |
| **Anti-coagulant medications** |  |
| Warfarin | B01AA03 |
| NOACs | B01AE07, B01AF01, B01AF02, B01AF03 |
| **Anti-platelet medications** |  |
| Aspirin | B01AC06, N02BA01 |
| Clopidogrel or Ticagrelor | B01AC04, B01AC30, B01AC24 |


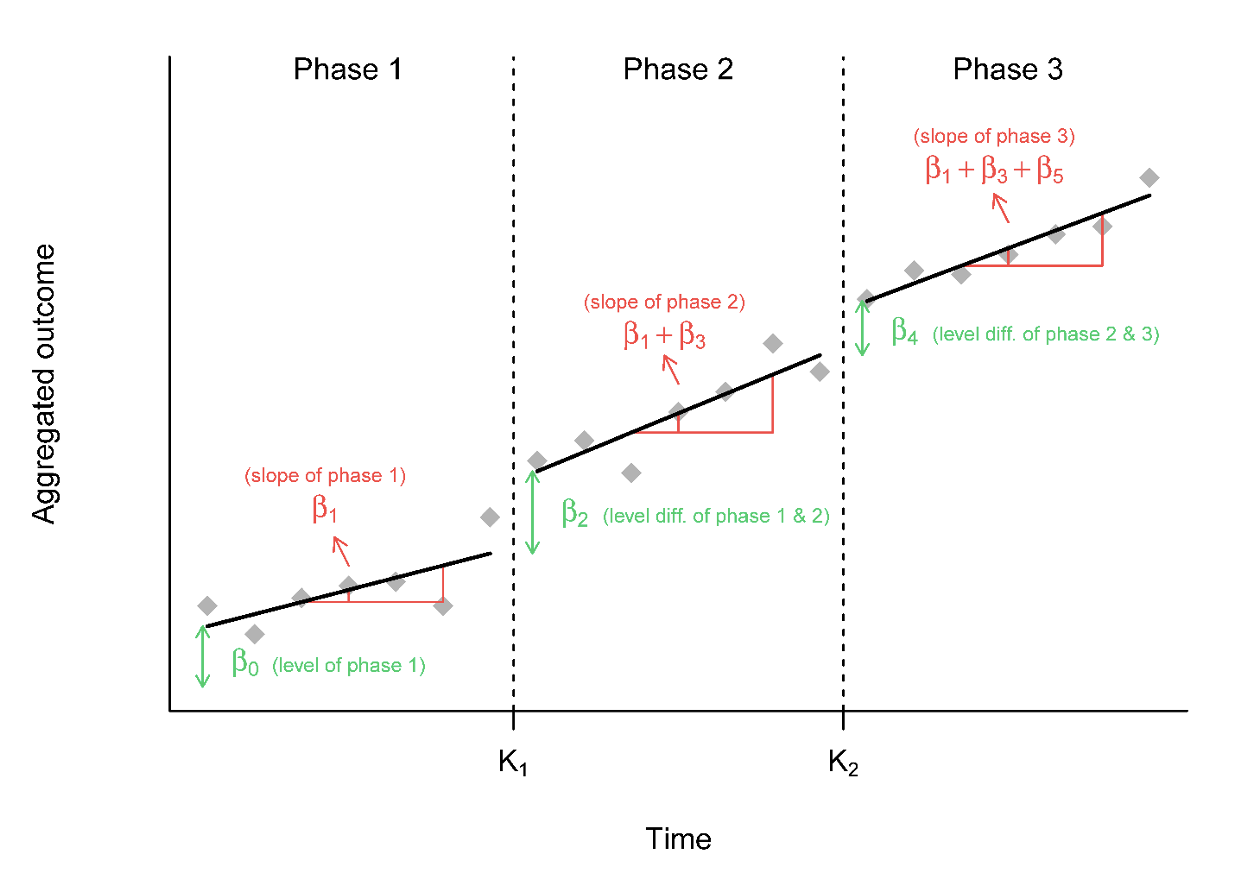


**Figure S1.** Scatter plot of interrupted time series of the aggregate outcome. The vertical line indicates three phases when RA medication was reimbursable by the Taiwan National Health Insurance program. Phase 1 (2000-2002) is the cDMARD era; Phase 2 (2003-2012) is the TNFi era; Phase 3 (2013-2017) is the OMA era.
