# Supplementary figures and images for: Trends of adverse events and mortality after DMARDs in patients with rheumatoid arthritis: Interrupted time‐series analysis
Source: Immun Inflamm Dis. 2022 Jun 20;10(7):e630. doi: 10.1002/iid3.630 (PMC9208285; doi:10.1002/iid3.630)

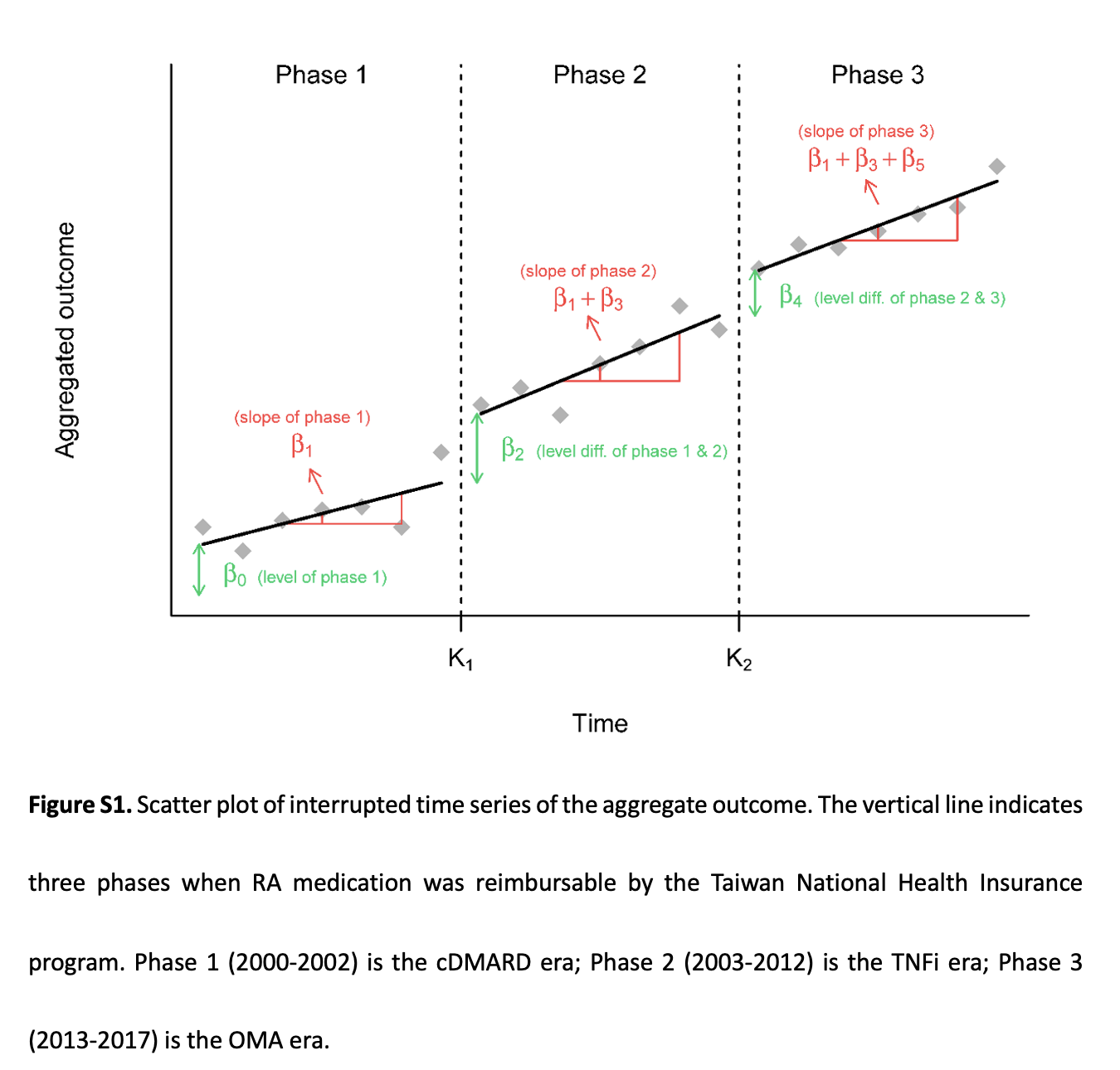

Supplement: Supplementary file 2 — Supplementary Information [file IID3-10-e630-s001.tiff]
